# Isolated Abdominal Wall Actinomycosis Associated with an Intrauterine Contraceptive Device: A Case Report and Review of the Relevant Literature

**DOI:** 10.1155/2010/340109

**Published:** 2010-08-12

**Authors:** Sinan Carkman, Volkan Ozben, Haydar Durak, Kagan Karabulut, Turgut Ipek

**Affiliations:** ^1^Department of General Surgery, Istanbul University, Cerrahpasa Medical School, Genel Cerrahi Anabilim Dali, Postal code 34098, Cerrahpasa, Fatih, Istanbul, Turkey; ^2^Department of Pathology, Istanbul University, Cerrahpasa Medical School, Patoloji Anabilim Dali, Postal code 34098, Cerrahpasa, Fatih, Istanbul, Turkey

## Abstract

Isolated abdominal wall actinomycosis in the presence of an intrauterine contraceptive device (IUCD) is extremely rare and only six such cases have been reported in the literature. We report a case where clinical and radiological examinations revealed a pseudotumor within the anterior abdominal wall. After being lost to follow-up, the patient presented two years later with the enlargement of the mass. The mass including the affected anterior abdominal wall was completely excised. The diagnosis of actinomycosis was established postoperatively by histopathological examination. Further questioning concerning her gynecological history revealed long-term use of the same IUCD. Surgical excision of the actinomycotic pseudotumour and removal of the IUCD followed by antibiotic therapy resulted in the full recovery of the patient.

## 1. Introduction

Actinomycosis is a rare subacute or chronic infectious disease resulting in suppurative and granulomatous inflammation. The disease is caused by anaerobic or facultatively anaerobic, acid-resistant Gram-positive bacteria of *Actinomyces species* [1]. In healthy subjects, *Actinomyces* is a part of normal flora of the oral cavity, gastrointestinal tract, and the genital tract. Healthy mucosa acts as a barrier to the spread of the organism, but tissues damaged by neoplasm, surgery, trauma, or foreign body allow multiplication and spread of actinomycosis [2, 3]. Prolonged use of intrauterine contraceptive devices (IUCD) in the pelvic region can account for actinomycotic infections ascending from the uterus. Actinomycosis of the abdominal wall in the presence of an IUCD without pelvic or intraperitoneal involvement is extremely rare [3–8] and poses a diagnostic challenge for clinicians, as it can be confused with malignancies [9]. In this present paper, we report a case of isolated abdominal wall actinomycosis caused by long-term use of an IUCD. To the best of our knowledge, only six cases were reported in the literature regarding primary abdominal wall actinomycosis in the presence of an IUCD.

## 2. Case Report

**Figure 1:**
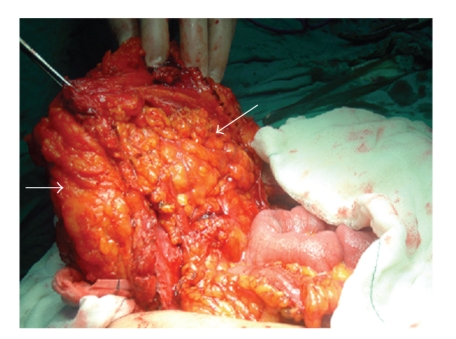
Complete excision of the actinomycotic anterior abdominal wall mass including the affected abdominal wall structures (left arrow) and the adjacent omentum (right arrow).

**Figure 2:**
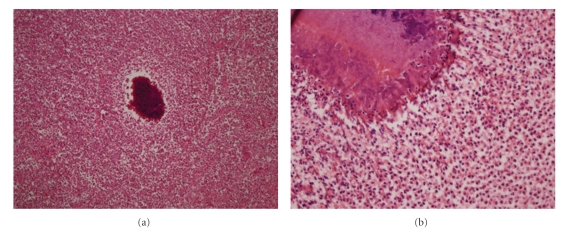
(a) Colonies of actinomyces with surrounding active chronic inflammatory cells and diffuse fibrosis (hematoxylin and eosin, x40), and (b) the same focus containing the filamentous colonies of actinomyces (x400).

A 48-year-old Caucasian woman presented with a painful abdominal wall mass in June, 2003. She did not have any history of trauma, surgery, or malignancy. Physical examination revealed a fixed and firm mass, about 5 cm in size, located around the umbilicus. The patient was afebrile. Laboratory parameters including erythrocyte sedimentation rate, complete blood count, C-reactive protein, and tumour markers were in normal values. Abdominal magnetic resonance imaging (MRI) showed an inhomogeneous mass composed of fibrous bands in the subcutaneous tissue in the periumbilical region, 6 × 5 cm in size, with no visceral involvement. An incisional biopsy was performed twice within a month under local anesthesia, where diffuse fibrosis and exudated suppurations were encountered. The histopathological examination indicated active chronic inflammatory changes, fat necrosis, and diffuse myofibroblastic activity corresponding to the diagnosis of an inflammatory pseudotumor. Microbiological examination and bacterial culture did not reveal any convincing findings. After initiating empirical antibiotic therapy with amoxicillin-clavulanic acid per oral, the patients was discharged, however, she was lost to medical follow up. In March 2005, she was readmitted to our clinic with the deterioration of her symptoms. Repeat MRI of the abdomen demonstrated the progression of the pseudotumor which reached a size of approximately 13 cm in diameter within the abdominal wall. *Surgical exploration was decided* on the basis of her medical history, previous histopathological, and the radiological findings. Following an abdominal transverse incision, intraoperative findings revealed a fibrotic mass of 12 × 13 cm within all layers of the anterior abdominal wall (Figure 1). The omentum was adherent to the parietal peritoneum underneath the mass. The lesion was excised including the affected muscles and the omentum. The resulting abdominal wall defect was closed by implantation of a 20 × 20 cm intraperitoneal polypropylene mesh (Prolene, Ethicon, Johnson & Johnson, Somerville, NJ, USA). Histopathological examination showed acute and chronic inflammatory changes due to actinomycotic colonies (Figure 2). Once the diagnosis was confirmed, a further questioning concerning her gynecological history revealed that she had been using the same IUCD without change for eight years. Gynecological examination was unremarkable except for an IUCD (Multiload Cu-375, Multilan, Organon, Oss, The Netherlands) which was removed consequently. Culture results from the IUCD revealed actinomycosis. She was prescribed a high-dose penicillin G (10 million units/day for three weeks) therapy and discharged on the seventh postoperative day without any complication. Oral penicillin was continued for further eight months. She recovered uneventfully and has been disease-free for four years. 

## 3. Discussion

Actinomyces bacteria are considered to be saprophytes in the oral cavity, throughout the gastrointestinal tract and the female genital tract. Actinomycosis is a chronic abscess-forming disease predominantly caused by *Actinomyces israelii*. Pathologic presentations of actinomycosis include cervicofacial (50% of cases), abdominal (20%), thoracic (20%), and pelvic involvement (15%) [10]. The destruction of mucosal barrier by trauma, operations, immunosuppression, and chronic inflammatory disease is recognized as predisposing factors for the penetration of the bacteria [9]. There has been a significant change in the epidemiology of the actinomycotic infections over the decades due to use of IUCDs [11]. Lunca et al. [3] reported that 75% of actinomycosis cases are seen in women, and 63% of the cases are associated with IUCD. 

 IUCDs have a traumatizing effect on endometrium, causing erosions that, in the presence of preexisting pelvic inflammatory disease or anaerobic infection, create a favorable environment for the development of actinomyces [12]. The presence of a long standing IUCD is a well-known risk factor for actinomycosis. There is a clear relationship between the risk of colonization and the duration of IUCD use [3, 5]. *A. israelii* is present in the reproductive tract of 1.6–44% of IUCD users and the incidence rises slightly after two years of IUCD use [13]. There is no doubt that long-term use of an IUCD represents a risk factor for pelvic actinomycosis and potentially for secondary dissemination to a distant site such as in the abdominal wall. The infection rarely disseminates by either lymphatics or the hematogenous route [2, 3, 10]. As in the presented case, the actinomycosis infection was associated with the prolonged use of IUCD (Multiload Cu-375) which has a normal life-span of three to five years. 

Histologically, suppurating abscesses with subsequent necrosis and dense fibrosis are usually seen in actinomycosis. The abscess cavities grow in size and have avascular thick walls. It initially creates dense adhesions with contiguous structures due to extensive fibrosis and in the late stages, can produce internal or external fistulae [11]. Besides the anterior abdominal wall, whereon localization is very rare, actinomycosis can affect the large intestine, liver and biliary tract, stomach, pancreas, greater omentum, and kidney [9]. Primary actinomycosis of the anterior abdominal wall has been reported only in 29 cases in the literature by 2010 [2–5, 9, 14–18]. However, the association between the use of IUCD and the occurrence of isolated abdominal wall actinomycosis has been sporadically reported. Since the first case described by Adachi et al. [8] in 1985, a Medline search of the medical literature revealed only six cases in total [3–8].

Actinomycosis has been called “the most misdiagnosed disease”, and it has been said that “no disease is so often missed by experienced physicians” [13]. Clinically, there is no evidence of specific symptoms related to actinomycosis in the anterior abdominal wall. In laboratory analysis, the dominating signs are anemia, leucocytosis, and positive inflammation markers. Ultrasonography, computed tomography, or MRI do not suffice to differentiate between actinomycosis and other inflammatory or neoplastic processes [2, 9]. Preoperative diagnosis is rarely established in approximately only 10% of cases, and the pathology can therefore be confused with other conditions such as neoplasia or another inflammatory cause [2, 3, 9]. It is also very difficult to isolate the bacterium from cultures. The culture medium should be strictly anaerobic and takes between 14 and 21 days to establish diagnosis. Such a specific culture request is not often made if the diagnosis is not suspected. Negative culture rate was reported to be 76% [3]. This is the reason why a wide primary resection including the surrounding tissue is inevitable [2, 9]. In the presented case, the diagnosis could not be made by the microbiological and histopathological examinations of the repeated incisional biopsies, which eventually resulted in the progression of the disease. Thus, the second operation involved a wide excision of the tumor with the affected muscles and the abdominal wall defect was closed by the implantation of an inlay prolene mesh. Ladurner et al. [2] also described a similar surgical procedure and subsequent implantation of an intraperitoneal mesh in a case with primary actinomycosis of the anterior abdominal wall. 

It is critical to repair abdominal wall defects with nonabsorbable meshes in the presence of infection. A study by van't Riet et al. [19] shows that the use of absorbable mesh is associated with an increased incidence of complications and mortality when compared to nonabsorbable mesh material. Polypropylene mesh is the most widely used nonabsorbable material for abdominal wall replacement and reinforcement due to its favorable characteristics such as durability, pliability, high-tensile strength, porosity, and good growth of fibroblasts into the mesh [19, 20]. As in the presented case with actinomycotic infection, the polypropylene mesh has not resulted in any complications over a four-year follow-up period.

Surgical treatment without antibiotic therapy is not always sufficient to achieve a cure of actinomycosis. When antibiotic therapy is combined with surgery, it is relatively simple to treat, and the cure rate is more than 90% [3, 10, 21]. Penicilin and tetracycline are both effective. Initial treatment should be parenteral penicillin G in high doses of 10–20 million units per day for two to four weeks and continued with oral penicilin V at a dose of 2–4 g/day. Because of the extensive necrosis and low vascularity, antimicrobial therapy should be continued until all signs of inflammation disappear, and this may take from several months to a year or more [9, 22]. In order to prevent recurrences, users of IUCD should be suggested to remove the intrauterine device. 

In conclusion, isolated actinomycosis of the abdominal wall in IUCD users is an extremely rare clinical entity. Preoperative diagnosis is difficult and rarely established. Long-term use of an IUCD represents a risk factor and should be removed in such clinical presentations. The combination of surgery and antibiotic therapy is curative in most of the cases.

## References

[1] Kaszuba M, Tomaszewska R, Pityński K, Grzanka P, Bazan-Socha S, Musiał J (2008). Actinomycosis mimicing advanced cancer. *Polskie Archiwum Medycyny Wewnetrznej*.

[2] Ladurner R, Bogner JR, Drosse I (2008). A rare case of primary actinomycosis of the anterior abdominal wall: diagnosis and treatment. *Hernia*.

[3] Lunca S, Bouras G, Romedea NS, Pertea M (2005). Abdominal wall actinomycosis associated with prolonged use of an intrauterine device: a case report and review of the literature. *International Surgery*.

[4] Polat I, Gungorduk K, Polat G, Yildirim G, Aslan H, Tekirdag AI (2008). Persistent subumbilical discharge associated with actinomycosis caused by intrauterine contraceptive device: a case report. *Archives of Gynecology and Obstetrics*.

[5] Çöl C, Çöl M, Albayrak L (2000). A case report of abdominal wall actinomycosis associated with prolonged use of an intrauterine device. *Antimicrobics and Infectious Diseases Newsletter*.

[6] Groot G, Rivers L, Smith T, Urbanski P, Boyle C (1991). Abdominal wall actinomycosis associated with use of an intrauterine device: a case report. *Canadian Journal of Surgery*.

[7] Pearlman M, Frantz AC, Floyd WS, Faro S (1991). Abdominal wall Actinomyces abscess associated with an intrauterine device: a case report. *Journal of Reproductive Medicine for the Obstetrician and Gynecologist*.

[8] Adachi A, Kleiner GJ, Bezahler GH, Greston WM, Friedland GH (1985). Abdominal wall actinomycosis associated with an IUD: a case report. *Journal of Reproductive Medicine for the Obstetrician and Gynecologist*.

[9] Filipović B, Milinić N, Nikolić G, Randelović T (2005). Primary actinomycosis of the anterior abdominal wall: case report and review of the literature. *Journal of Gastroenterology and Hepatology*.

[10] Owen K, Flannery MT, Elaini AB, Rivera J (2004). Actinomycotic tumor of the abdominal wall. *Southern Medical Journal*.

[11] Biyani DK, Denley H, Hill J, Watson AJM (2007). IUCD induced abdomino-pelvic actinomycosis presenting as acute large bowel obstruction. *Journal of Obstetrics and Gynaecology*.

[12] Waaddegaard P, Dziegiel M (1988). Actinomycosis mimicking abdominal neoplasm. Case report. *Acta Chirurgica Scandinavica*.

[13] Ko T-L, Li Y-T, Chu Y-C (2007). An uncommon case of pelvic and abdominal wall mass: presumed pelvic actinomycosis. *Taiwanese Journal of Obstetrics and Gynecology*.

[14] Urbánek S, Kliment LML, Holub Z (2005). Fistula as a complication of pelvic actinomycosis—two case reports. *Ceska Gynekologie*.

[15] Hefny AF, Joshi S, Saadeldin YA, Fadlalla H, Abu-Zidan FM (2006). Primary anterior abdominal wall actinomycosis. *Singapore Medical Journal*.

[16] Yi F, Prasad S, Sharkey F, Kahlenberg M (2008). Actinomycotic infection of the abdominal wall mimicking a malignant neoplasm. *Surgical Infections*.

[17] Mueller MC, Ihrler S, Degenhart C, Bogner JR (2008). Abdominal actinomycosis. *Infection*.

[18] Gómez-Ramírez J, Martín-Pérez E, Alcaide B, Martín-Álvarez JL, Larrañaga E (2009). Primary abdominal wall actinomycosis. *Cirugia Espanola*.

[19] van’t Riet M, de Vos van Steenwijk PJ, Bonjer HJ, Steyerberg EW, Jeekel J (2007). Mesh repair for postoperative wound dehiscence in the presence of infection: is absorbable mesh safer than non-absorbable mesh?. *Hernia*.

[20] McNeeley SG, Hendrix SL, Bennett SM (1998). Synthetic graft placement in the treatment of fascial dehiscence with necrosis and infection. *American Journal of Obstetrics and Gynecology*.

[21] Das N, Lee J, Madden M, Elliot CS, Bateson P, Gilliland R (2006). A rare case of abdominal actinomycosis presenting as an inflammatory pseudotumour. *International Journal of Colorectal Disease*.

[22] Kaya M, Sakarya MH (2007). A rare cause of chronic abdominal pain, weight loss and anemia: abdominal actinomycosis. *Turkish Journal of Gastroenterology*.

